# Items in Brief

**Published:** 1873-06

**Authors:** 


### Items in Brief.

—The Vienna mixture for anæsthesia contains four parts by measure of ether to one of chloroform. These mixed anæsthetics have been more or less in use since 1849, but were early said by Snow, who was a thorough experimenter in anæsthesia, to be most objectionable. He stated that ether evaporates in one-sixth the time that chloroform does and consequently when the most caution in the administration is desired, chloroform alone is the acting anæsthetic. Dr. T. Spencer Wells has lately reiterated this assertion. It is irregular, slow, and disagreeable in its action, and though it has been very extensively used with good results it has been followed by fatal effect.—Dr. D. W. Yandell, in the *American Practitioner*, has given account of five cases of tertiary syphilis treated with bromide of quinine in doses of from three to five grains, three times daily.—Carbazotate of ammonium is a new article recommended in intermittent fever by Dr. Dujardin-Beaumetz, who says its action is analogous to that of sulphate of quinia.

—Pasteur, in a new series of experiments upon fermentation, claims that pure grape juice when exposed to the air or oxygen never alone ferments but requires the particles of dust, germs or woody fibre of the fruit or vine, to induce the alcoholic change. He states that there are two classes of organisms in nature, those which live upon oxygen free or combined, and those which are germs of ferment which require oxygen derived from a compound like carbonic acid and to which free oxygen or that otherwise combined, acts as a poison. Fruits ripen in the air when picked, without fermentation, but enclosed in an atmosphere of carbonic acid completely loses its vitality and undergoes perfect alcoholic fermentation.

—The new method in photography, called gelatine process, by which photograph printing is rendered independent of the sun's direct assistance— a thousand copies being struck off by the use of ordinary printer's ink in the time occupied in executing a few dozen by means of sun-printing—has proved a wonderful success, and bids fair to supersede lithography and in many cases steel engraving. This improved process has just been brought to a state of great perfection by M. Albert, of Munich, Bavaria, who has made it possible to print several thousand impressions from the same plate, and at a comparatively small cost. A description of this method, in its various chemical and mechanical details, would occupy much space. Suffice it to say that, by means of it pictures from the cheaply produced gelatine plates can be printed at a cost not exceeding that of an ordinary lithograph. In printing, a common lithographic press is used, and the operation is the same as in the production of lithographs; indeed, the pictures combine the qualities of the lithograph with the delicacy of the steel engraving and the accuracy of the photographer.—*Am. Newspaper Reporter.*

—Claude Bernard is still prosecuting his studies on the subject of the formation and disposition of sugar. He has recently shown to the Society of

Biology that when sugar is injected into the veins it is not at all absorbed, but is hurried out of the economy in the urine; but when it is carried into alimentary canal it is changed by a ferment present in the small intestine, taken up by the liver and fixed as glycogen.—*The Clinic.*——Dr. Dills, of Carlisle, Ky., (*The Clinic*, March 29th,) was called to attend a colored girl in labor, aged eleven years and nine months, and found a vertex presentation, first position; the pelvis was large and roomy, labor progressed rapidly, and a living child weighing nine and a half pounds was the result. The girl had never menstruated. Lobstein and Canes each relates a case in which menstruation commenced in and continued regularly after the second year of age; in one of these cases the girl conceived in her eighth year.—*Med. Record.*

--The Pennsylvania Legislature has lately given one hundred thousand dollars to the Jefferson Medical College of Philadelphia, as a contribution to the fund for building a college hospital.——Philadelphia has 699 regular physicians of which 50 are on the retired list, and has a population of 674,022.——The Boylston medical prizes have been awarded to T. M. Ratch for essay on "The Emigration of the White Corpuscle in Inflammation," and to Walter Ela for essay on "Fractures of the Elbow-joint."——Drs. Fordyce Barker and T. Gailard Thomas, of New York, were elected honorary members of the Obstetrical Society of London, at a late meeting.——Dr. Chas. P. Russell, Registrar of Records in the New York City Health Department, states that since March 10th, 1871, when John Thomas Rosenville was hung, 104 homicides been committed in that city; of which only one, Wm. Foster, March 22st, 1873, was executed.——New Haven is attempting to raise a fund of $30,000 to establish a training-school for nurses at the Connecticut State Hospital in that city. Success to so worthy an object.—*Reporter.*——A movement is being made to erect a statue to the celebrated Italian anatomist, Bartholomew Eustachius, by the people of his native place, San Severino.——A Massachusetts man lately sold seventy-three dozen patent medicine bottles, all of which had been emptied in his own family.——Mr. John Hopkins, of Baltimore, recently deeded to trustees thirteen acres of land in that city for the relief of indigent sick and orphans. At a meeting of the board of trustees March 11th, they were notified by Mr. Hopkins that he had further dedicated $2,000,000 of property for the support and maintenance of the hospital. The hospital buildings will be on a magnificent scale and their erection will be begun in the spring of 1874.—*The Clinic.*——The death of Hugh L. Hodge, M. D., L.L. D., of Philadelphia, for thirty years professor of Obstetrics in the University of Pennsylvania, occurred February 26th. He was the author of "Hodge's System of Obstetrics" and "Diseases Peculiar to Women," and was widely known at home and abroad. He was in active work till just before his death.——Baron Justus Von Liebig, died at Munich, on Friday, April 18th, in the seventy-first year of his age.——The death of Baker Brown, F. R. C. S., has lately been announced.

